# What is the optimal recall period for verbal autopsies? Validation study based on repeat interviews in three populations

**DOI:** 10.1186/s12963-016-0105-1

**Published:** 2016-10-18

**Authors:** Peter Serina, Ian Riley, Bernardo Hernandez, Abraham D. Flaxman, Devarsetty Praveen, Veronica Tallo, Rohina Joshi, Diozele Sanvictores, Andrea Stewart, Meghan D. Mooney, Christopher J. L. Murray, Alan D. Lopez

**Affiliations:** 1 Institute for Health Metrics and Evaluation, University of Washington, 2301 Fifth Ave., Suite 600, Seattle, WA 98121 USA; 2 University of Queensland, School of Public Health, Level 2 Public Health Building School of Public Health, Herston Road, Herston, QLD 4006 Australia; 3 The George Institute for Global Health – India, Unit No. 301, Second Floor, ANR Center Road No.1, Banjara Hills, Hyderabad, Telangana 500 034 India; 4 Research Institute for Tropical Medicine, Corporate Ave, Muntinlupa City, 1781 Philippines; 5 The George Institute for Global Health – Australia, Level 10, King George V Building, 83-117 Missenden Rd, PO Box M201, Camperdown, NSW 2050 Australia; 6 University of Melbourne, School of Population and Global Health, Building 379, 207 Bouverie St., Parkville, 3010 VIC Australia

**Keywords:** Verbal autopsy, Cause of death, Recall period

## Abstract

**Background:**

One key contextual feature in Verbal Autopsy (VA) is the time between death and survey administration, or recall period. This study quantified the effect of recall period on VA performance by using a paired dataset in which two VAs were administered for a single decedent.

**Methods:**

This study used information from the Population Health Metrics Research Consortium (PHMRC) Study, which collected VAs for “gold standard” cases where cause of death (COD) was supported by clinical criteria. This study repeated VA interviews within 3–52 months of death in PHMRC study sites in Andhra Pradesh, India, and Bohol and Manila, Philippines. The final dataset included 2113 deaths interviewed twice and with recall periods ranging from 0 to 52 months. COD was assigned by the Tariff method and its accuracy determined by comparison with the gold standard COD.

**Results:**

The probability of a correct diagnosis of COD decreased by 0.55 % per month in the period after death. Site of data collection and survey module also affected the probability of Tariff Method correctly assigning a COD. The probability of a correct diagnosis in VAs collected 3–11 months after death will, on average, be 95.9 % of that in VAs collected within 3 months of death.

**Conclusions:**

These findings suggest that collecting VAs within 3 months of death may improve the quality of the information collected, taking the need for a period of mourning into account. This study substantiates the WHO recommendation that it is reasonable to collect VAs up to 1 year after death providing it is accepted that probability of a correct diagnosis is likely to decline month by month during this period.

**Electronic supplementary material:**

The online version of this article (doi:10.1186/s12963-016-0105-1) contains supplementary material, which is available to authorized users.

## Background

Cause of death (COD) data are essential for informed planning in the health sector. Mortality statistics are a key input into tracking changes in the burden of disease in a population over time. This information can then be used to set, or reset, priorities for health interventions, monitor the efficacy of public health programs, and inform the allocation and distribution of limited resources within the health sector. Unfortunately, reliable cause of death information is least available in low-resource settings where arguably it is most crucial to public health research and decision-making [1]. Such crucial gaps in the knowledge base make it difficult to ensure informed decision-making for public health policy [2]. Increasingly, verbal autopsy (VA) methods are being seen as the most cost-effective means for filling this information gap. In addition, in places with limited or no Civil Registration and Vital Statistics Systems (CRVS), the use of VA has been recommended as part of CRVS system improvement efforts.

A VA is a questionnaire administered to the caregivers or family members of a person who has died (the decedent) to elicit specific signs and symptoms that occurred in the period before death. VAs have been used in demographic surveillance sites and in studies of the epidemiology of disease for over 40 years [3–7]. Modern VA instruments (VAIs) include those used by the World Health Organization (WHO) [8] and the Population Health Metrics Research Consortium (PHMRC) [9].

As a survey methodology, there is reason to believe that recall bias may affect the validity of VAs [10–12]. Currently, WHO recommends that, after a period of mourning, the VA be conducted as soon as possible; recalls of more than 1 year should be interpreted with caution [13]. There is a paucity of evidence to support such a recommendation, which appears to be based on an unpublished study from 2001 that showed no difference in sensitivity and specificity of VA for recall periods up to 3 years (Chandramohan D: Verbal autopsy tools for adult deaths. PhD Thesis. London School of Hygiene and Tropical Medicine. University of London, 2001, unpublished) [14]. A more recent study in Burkina Faso and Indonesia examined the agreement between verbal autopsy administered at different intervals after death [15]. Both of these studies estimated reliability – i.e., agreement between two VAs administered at different time intervals – but not accuracy: whether the results were concordant with a gold standard COD at different recall periods.

The standard instruction for collecting VAs is to allow for a period of mourning after death and then to administer the VA as soon as is practicable. Recall bias is not a major issue in well-functioning CRVS systems with continuous registration of deaths or in Demographic Surveillance Sites with short intervals between survey rounds. It is, however, an issue in the collection of VAs in cross-sectional surveys, such as national censuses or Demographic and Health Surveys, where there are long intervals between survey rounds.

In this study we aimed to quantify the effect of the recall period on VA diagnostic performance using a dataset in which the true cause of death was known with a high degree of certainty [9] and applying the most recent version of an automated diagnostic VA method (Tariff 2.0), for assigning COD from VA data [16]. We did so by using a paired dataset in which two VAs were administered for a single decedent with recall periods ranging from 6 days to 52 months. This subset of the PHMRC gold standard dataset [9] comprised 2113 decedents with known COD in Bohol and Manila in the Philippines, and in Andhra Pradesh in India. We aimed to make an evidence-based recommendation about the maximum length of time between death and administration of the VA interview that would still maintain high-quality COD predictions obtained from automated VA analysis.

## Methods

### Data

The general methodology of the PHMRC study has been described in detail elsewhere and is summarized here for convenience [9]. Gold Standard (GS) clinical diagnostic criteria for hospital deaths were first established for a list of 34 adult, 21 child, and six neonatal causes including stillbirths (see Additional file 1 for the list of target causes for the VA). Deaths with hospital records fulfilling the GS criteria were identified in each of the sites. Families were interviewed about the events leading to each of these deaths using the PHMRC VAI, which has separate modules for adults, children, and neonates. Interviewers were blinded to the COD assigned in the hospital. The full PHMRC database contains 12,535 verbal autopsies with GS diagnoses (7846 adults, 2064 children, 1620 neonates, and 1005 stillbirths).

This study was based on VAs of deaths in the PHMRC GS validation dataset that occurred during 2007 and 2008 in Bohol, Manila, and Andhra Pradesh. For each death a gold standard cause of death had been identified and a verbal autopsy interview been collected during the PHMRC study between 6 days and 5 months after the death. In this paper, we will refer to these as first-round VAs. We revisited a subset of these and collected a second VA from the same families (second-round VAs). When revisiting households the same respondent was interviewed again; however, if this was not a possible a different family member was interviewed for the second VA.

This study had a convenience sample aiming to explore the question of time between death and VA interview and accuracy. The symptom recall study revisited families who had provided verbal autopsies for the Population Health Metrics Research Consortium gold standard verbal autopsy validation study (PHMRC study). Data collection as an extension of the PHMRC study occurred 3–20 months after death in Bohol, Manila, and Andhra Pradesh for second-round VAs. The samples were based on all cases that had been studied over a calendar year in the PHMRC study which could still be identified in the community.

Further data collection funded by the Australian National Health and Medical Research Council (NHMRC) occurred in Bohol, where additional second-round VAs were collected 18–52 months after death. For clarity, we describe the two separate data collection periods for second-round VAs as Bohol (1) and Bohol (2) from the PHMRC and NHMRC grants, respectively. The Bohol (2) cases were selected from the calendar year 2007–2008 in an attempt to test the validity of longer periods of recall.

The methods of this study were approved by the Internal Review Boards of the University of Washington, Seattle, WA, USA; School of Public Health, University of Queensland, Australia; University of Sydney, Australia; National Institute of Public Health, Mexico; Research Institute for Tropical Medicine, Alabang, Metro Manila, Philippines; Muhimbili University, Tanzania; Public Health Laboratory Ivo de Carneri, Tanzania; and CSM Medical University, India. All data were collected with informed consent from participants.

### VA cause of death assignment

COD was assigned using Tariff 2.0, the VA analytic method recommended for use in routine mortality surveillance systems based on the comparative performance of existing methods [17]. The Tariff 2.0 method is a simple additive algorithm that creates a score, or tariff, for each questionnaire-symptom pair [16, 18]. We assigned a COD for each of the two verbal autopsies collected for each of the 2113 deaths.

### Variables

We created a series of variables as the basis for logistic regression. We generated a binary indicator for “correct assignment”; this was coded as zero if the prediction (i.e., diagnosis) from applying the Tariff method to the VA differed from the gold standard COD, and one when it was identical. A continuous variable labelled “time” was defined as the number of months between death and administration of the verbal autopsy. Binary indicators were also created for the different sites/populations (Andhra Pradesh, Bohol (1), Bohol (2), and Manila) and the different modules (neonate, child, and adult).

### Statistical analysis

The aim of the analysis was to quantify the effect of recall period on the probability of correctly assigning the COD using Tariff 2.0. The effect of recall period on deriving the correct cause assignment (assessed against the gold standard) was measured using a logistic regression framework as specified in Equation 1, controlling for confounding due to site of data collection and survey module (adult, child, or neonate). The predicted probabilities of correct assignment were then calculated from these models. The data were also clustered on the individual to control for the paired (i.e., non-independent) observations. All analyses were done using Stata 13.1 [19].

Each module has a different set of causes associated with it (see Additional file 1). The probability of correct cause assignment varies among modules. Thus the probability of a neonatal death, with six possible causes, having a cause correctly assigned by random chance is much higher than for a child (21 possible causes) of for an adult (34 possible causes). Recall period squared was used as a covariate because of the possibility of a non-linear association between time and correct cause of death assignment. We also relaxed the assumption of independence between observations for verbal autopsy diagnoses from the same decedent. Because each individual death in the dataset has two verbal autopsies, a correct assignment was significantly more likely in the second VA if it had also been selected in the first VA (correlation coefficient of 0.485). Merely setting a fixed effect that differentiated between the first versus the second VA would have detracted from the effect for the true predictor of interest: namely the time between the death and the interview. We therefore employed a clustered sandwich variance estimator [20, 21] using the logistic command in Stata for each regression. Using this command with a cluster in Stata is basically doing a generalized equation model using sandwich variance which relaxes the assumption of independence of two VAs from the one decedent.

We also controlled for data collection site: Andhra Pradesh, Manila, Bohol (1), and Bohol (2). Differences between the sites captured by this covariate are likely to include variations in data collection procedures (despite using a standardized protocol), cultural differences, and cause composition of deaths at different sites (Regression 1).

*Regression 1:*

$$ logit\left( correct\  assignment\right)={\upbeta}_0+{\upbeta}_1\; recall\  period+{\upbeta}_2{\left( recall\  period\right)}^2+{\upbeta}_3\; site+{\upbeta}_4\; module $$

To see if our results were robust, we conducted a sensitivity analysis which treated recall period as a series of categorical variables. Regression 2 uses the recall period of 0–2 months as a reference as compared to a recall period of 3–11 months and recall period ≥ 12 months.

*Regression 2:*

$$ logit\left( correct\  assignment\right)={\upbeta}_0+{\upbeta}_1\; recall\  period\ 3-11\  months+{\upbeta}_2\; recall\; period\ge\ 12\; months + {\upbeta}_3\; site+{\upbeta}_4\; module $$

In a third regression model, treating also the recall period as categorical variables, we used the recall period of 0–2 months as a reference as compared to a recall period of ≥3 months:

*Regression 3:*

$$ logit\left( correct\  assignment\right)={\upbeta}_0+{\upbeta}_1\; recall\  period\ge 3 months+{\upbeta}_2\; site+{\upbeta}_3\; module $$

In a secondary analysis we controlled for different characteristics of the respondent including the type of their relationship with the decedent, education, and sex. This was done within the same logistic regression framework, controlling for collection site and module and using a sandwich estimator to relax the assumption of independence of VAs from the same decedent. This analysis was stratified by module because the type of relationship between respondent and decedent varied with the age of the decedent. As a sensitivity analysis we also performed the three regressions using only adult decedents.

## Results

A total of 4226 verbal autopsies were collected for 2113 decedents in the three separate sites. Data collected from Bohol at the two different time periods are tabulated separately. Table 1 shows sample sizes of the sites and average recall periods for the first and second round of VA data collection. The mean interval between death and the first of the two interviews was 1.84 months. The mean between death and the second interview was 17.17 months; these data were skewed to the left because of the long delay (40 months) before the collection of data in Bohol (2). Relationship of respondents to decedent is presented in Additional file 2. Of the 2113 VAs, 1813 (86.7 %) had the same respondent for both interviews. More deaths in adults (1394) were studied than were deaths in children (349) or neonates (370). One hundred VAs had an indeterminate COD (37, 18, and 45 for adult, child, and neonatal modules, respectively) with the Tariff Method.

**Table 1:** Number of decedents by site and module and average recall period in months by site with standard deviation in parentheses

	Andhra Pradesh	Bohol (1)	Bohol (2)	Manila	Total
Adult	657	235	312	190	1394
Child	203	45	42	59	349
Neonate	157	69	107	37	370
Total	1017	349	461	286	2113
Recall period Round 1 ^a^	1.72 (0.76)	0.85 (0.49)	2.66 (0.58)	2.16 (0.57)	1.84 (0.87)
Recall period Round 2 ^a^	9.91 (2.22)	12.09 (3.57)	40.60 (4.03)	11.40 (3.52)	17.17 (12.79)
Survey dates Round 1	1 May 2009–30 Apr 2010	6 Jan 2009–30 Jan 2010	30 Jul 2007–24 Jul 2008	8 Jan 2009–30 Mar 2010	30 Jul 2007–30 Apr 2010
Survey dates Round 2	18 Feb 2010–16 Aug 2010	1 Mar 2010–28 Jul 2010	23 Nov 2010–13 Oct 2011	3 Mar 2010–30 Jul 2010	18 Feb 2010–13 Oct 2011

^a^ Mean (SE)

Tables 2, 3 and 4 shows combined and separate results from the two survey rounds and summarizes recall periods used in Regressions 2 and 3: 3251 VAs were collected in the first 12 months after death, and 975 VAs thereafter.

**Table 2:** Number of verbal autopsies by recall period and by site

Recall period	Andhra Pradesh	Bohol (1)	Bohol (2)	Manila	Total
0–2 months	952	347	300	259	1858
3–11 months	925	138	161	169	1393
12–23 months	157	213	0	144	514
24–35 months	0	0	61	0	61
≥36 months	0	0	400	0	400
Total	2034	698	922	572	4226

**Table 3:** Number of verbal autopsies by recall period and by site for round 1 VAs

Recall period	Andhra Pradesh	Bohol (1)	Bohol (2)	Manila	Total
0–2 months	952	347	300	259	1858
3–11 months	925	138	161	169	1393
12–23 months	157	213	0	144	514
24–35 months	0	0	61	0	61
≥36 months	0	0	400	0	400
Total	2034	698	922	572	4226

**Table 4:** Number of Verbal Autopsies by recall period and by site for round 2 VAs

Recall period	Andhra Pradesh	Bohol (1)	Bohol (2)	Manila	Total
0–2 months	0	0	0	0	0
3–11 months	860	136	0	142	1138
12–23 months	157	213	0	144	514
24–35 months	0	0	61	0	61
≥36 months	0	0	400	0	400
Total	1017	349	461	286	2113

**Fig. 1:**
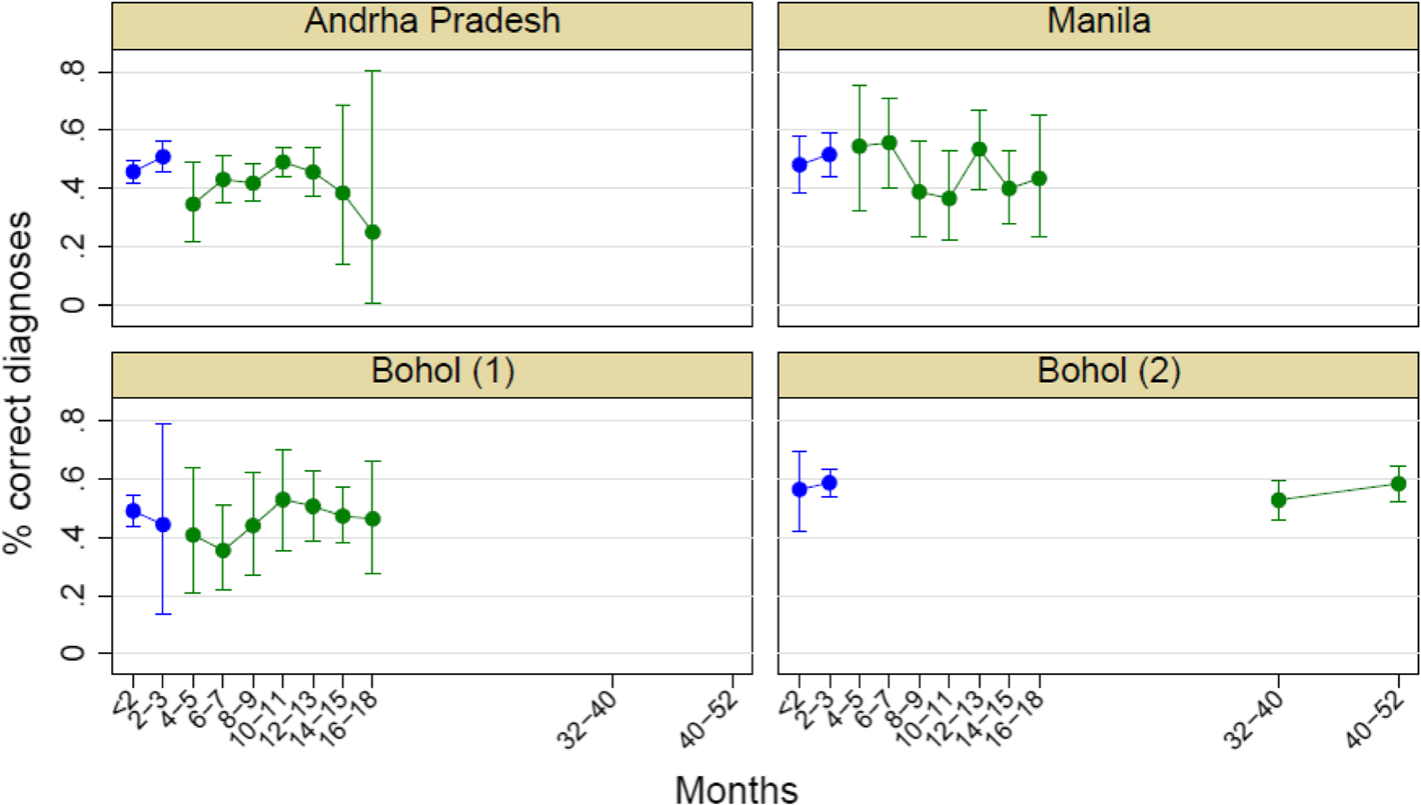
Simple concordance as a function of time from death to verbal autopsy administration, by site and VA survey round. VA survey round 1 is *blue* and VA survey round 2 is *green*

Simple (i.e., unadjusted) concordance and kappa by site and module for the 4226 VAs are summarized in Table 5. Mean concordance (i.e., average level of agreement between the gold standard cause assignment and that predicted by Tariff 2.0) for different recall periods across the four sites/populations was 0.491 overall, 0.470 for adults, 0.451 for children, and 0.609 for neonates. Mean kappa was 0.470 overall, 0.445 for adults, 0.408 for children, and 0.512 for neonates. Figure 1 shows simple concordance (or the percent of individual diagnoses that were correct) by the period since death, in months, for each site.

**Table 5:** Concordance and kappa between Tariff COD and Gold Standard COD by module and site

	Concordance (95 % CI)	Kappa (95 % CI)
Adult	0.470 (0.451, 0.488)	0.445 (0.437, 0.453)
Child	0.451 (0.414, 0.488)	0.408 (0.388, 0.427)
Neonate	0.609 (0.574, 0.645)	0.512 (0.478, 0.546)
Andhra Pradesh	0.463 (0.441, 0.485)	0.441 (0.432, 0.449)
Bohol (1)	0.476 (0.439, 0.513)	0.446 (0.429, 0.463)
Bohol (2)	0.570 (0.538, 0.603)	0.541 (0.525, 0.557)
Manila	0.481 (0.440, 0.522)	0.456 (0.438, 0.473)
Round 1	0.505 (0.484, 0.527)	0.485 (0.476, 0.494)
Round 2	0.477 (0.455, 0.498)	0.456 (0.447, 0.464)
Overall	0.491 (0.476, 0.506)	0.470 (0.464, 0.476)

**Fig. 2:**
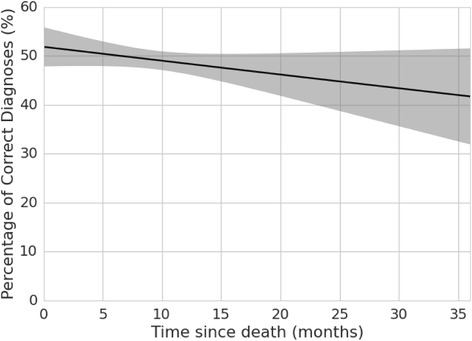
Probability of correct assignment as a function of time from death to verbal autopsy administration

Table 6 summarizes the effect of recall period on diagnostic accuracy using the three logistic regression frameworks. Regression 1 shows an odds ratio of 0.989 (95 % CI 0.974, 1.004) for recall by month. Estimating the probabilities of correct assignment from logistic regression model, this corresponds to a probability of correct assignment decreasing by 0.55 % per month (Fig. 2). There is no suggestion of a non-linear trend. This result takes into account higher levels of accuracy in Bohol (2) (OR = 1.492) and in neonates (OR = 1.729). Regression 2 shows an odds ratio of 0.922 (95 % CI 0.822, 1.034) for the period 3–11 months after death and of 0.917 (955 CI 0.799, 1.052) for the period ≥ 12 months. In regression model 3, the odds ratio of correct assignment for VAs collected 3 or more months after the dead was 0.92 (95 % CI 0.837, 1.011) as compared with those collected in the first 2 months. Estimating the probability of correct diagnosis from logistic regression 2, this implies that the probability of a correct diagnosis in VAs collected 3–11 months after death will, on average, be 95.9 % of that in VAs collected within 3 months of death. The probability of a correct diagnosis in VAs collected ≥ 12 months after death will, on average, be 95.6 % of that in VAs collected within 3 months of death (see Table 7). We also examined the recall time within each age group. Results are presented for adults, the only group with a large enough sample size for this analysis, in Additional file 3. For adults the results are consistent than the ones found for the whole population.

**Table 6:** Odds ratios from logistic regressions 1, 2, and 3 showing the effects of recall period on correct assignment for verbal autopsy pairs

Covariates	Regression 1	Regression 2	Regression 3
	OR (95 % CI)	OR (95 % CI)	OR (95 % CI)
	*N* = 4226	*N* = 4226	*N* = 4226
Recall period (months)	0.989 (0.974, 1.004)		
Recall period (months^2^)	1.000 (1.000, 1.001)		
Recall period 0–2 months (reference)			
Recall period 3–11 months		0.922 (0.822, 1.034)	
Recall period ≥ 12 months		0.917 (0.799, 1.052)	
Recall period ≥ 3 months			0.920 (0.837, 1.011)
Andhra Pradesh (reference)			
Manila	1.096 (0.873, 1.377)	1.092 (0.868, 1.373)	1.091 (0.868, 1.370)
Bohol (1)	1.026 (0.831, 1.267)	1.024 (0.826, 1.268)	1.022 (0.828, 1.262)
Bohol (2)	1.492 (1.202, 1.851)	1.498 (1.229, 1.827)	1.495 (1.236, 1.809)
Adult (reference)			
Child	0.965 (0.780, 1.194)	0.966 (0.780, 1.195)	0.966 (0.781, 1.195)
Neonate	1.729 (1.411, 2.119)	1.733 (1.414, 2.124)	1.733 (1.414, 2.124)

**Table 7:** Post estimation predicted correct assignment by time while holding all other covariates at their mean

Regression 1		Regression 2		Regression 3	
	Predicted correct assignment and (95 % CI) ^a^		Predicted correct assignment and (95 % CI) ^a^		Predicted correct assignment and (95 % CI) ^a^
Recall period 0–2 months	0.503 (0.480, 0.526)	Recall period 0 months	0.518 (0.479, 0.558)	Recall period 0–2 months	0.503 (0.480, 0.526)
Recall period 3–11 months	0.483 (0.456, 0.510)	Recall period 6 months	0.501 (0.479, 0.524)	Recall period ≥ 3 months	0.482 (0.461, 0.503)
Recall period ≥ 12 months	0.481 (0.449, 0.514)	Recall period 12 months	0.484 (0.463, 0.505)		
		Recall period 18 months	0.467 (0.431, 0.504)		
		Recall period 24 months	0.450 (0.394, 0.507)		
		Recall period 30 months	0.434 (0.356, 0.511)		
		Recall period 36 months	0.417 (0.319, 0.514)		

^a^ Predicted probabilities of correct assignment estimated from the logistic regression models including as covariates recall period, study site, and age module

## Discussion

The fundamental objective of this research was to generate an empirically based recommendation for the optimal recall period, or time, between death and VA interview, based on an analysis of repeat interviews of families for which a gold standard cause of death was known.

The first regression equation indicates that the probability of a correct diagnosis declines by 0.55 % per month. This means that the probability of a correct diagnosis in VAs collected in the third month after death would be 98.3 % of that in the first month and in the fourth month after death, 97.8 %. We used the second and third regression equations as a sensitivity analysis. We assumed best practice to be collection of VAs in the first 3 months after death and examined decline. The equations indicate, first, that the probability of a correct diagnosis in VAs collected between the fourth and twelfth months after death would be 95.9 % that of one collected the first 3 months after death, but with fairly wide limits of confidence that include the null value. This is consistent with a monthly decline of 0.55 % probability of correct diagnosis. The sensitivity analysis also indicates that there was little or no decline in accuracy for recall periods ≥ 12 months. However the evidence for lack of decline during this period is less firm because it conflicts with the evidence from the first regression equation of a monthly decline in accuracy of 0.55 %. Far fewer VAs were collected at 12 months or more than in the 12 months after death, affecting the precision of our estimates. On the other hand, this lack of decline is consistent with findings from the study which showed no difference in sensitivity and specificity of VA for recall periods up to 3 years [14].

Clearly, recall period is only one factor among many affecting the accuracy of COD assignment from VAs. We note the variation between sites and between modules. In considering variation by site we need to consider variation in factors affecting the interview itself: language, norms and biomedical concepts, and type of respondents and interviewers [14]. We should also consider variation in the cause composition of mortality and of the accuracy of Tariff Method by cause [16]. In considering variation by module we need to consider the length of the list of causes for the module and the accuracy of Tariff for specific causes.

It is important to keep in mind that deaths included in this study took place in health facilities. It should also be noted that participating in a VA interview after a death of a relative/friend raises different emotions for respondents, and may refresh memories, trigger the search for further information, or cause the reinterpretation of the events leading to the death. Therefore, it is difficult to assume the conditions for answering a first and second VA interview about the same death are the same, especially if the informant is the same. This process can introduce variations in responses that, although difficult to quantify, should be taken into account.

## Conclusions

We conclude that accuracy of VAs declines at a rate of 0.55 % per month in the first year after death. Findings of this study suggest that collecting information in the first 3 months after the death –taking into account a period of mourning – may improve the quality of the information. However, the probability of a correct diagnosis in VAs collected between four and 12 months after death will, on average, be 95.9 % of those collected in the first 3 months.

So far, decisions about the best recall time for VA collection have been based on empirical or practical considerations, without a systematic and evidence-based approach to make such decisions. The results of this study have, therefore, important practical implications for the collection of VAs, both in research settings or reinforcing civil and vital registration systems. This study substantiates the WHO recommendation that it is reasonable to collect VAs up to 1 year after death providing it is accepted that accuracy is likely to decline month by month during this period. We have some evidence to suggest that the rate of decline decreases after the first year, but this is not strong enough to change the WHO recommendation.

Verbal autopsy is increasingly being considered for routine application in civil registration and vital statistics systems, yet many aspects related to best implementation practices remain unclear. Key among these is the optimal period in which to administer a verbal autopsy after death, and how longer waiting periods might affect the accuracy of diagnosis. By providing empirical evidence on this important issue, we hope to better inform decisions about verbal autopsy use in countries and promote its wider application to generate policy-relevant information on leading causes of death.

## Additional files

Additional file 1: List of target causes for the VA for adults, children and neonates. (DOCX 12 kb)
Additional file 2: Summary of the sample size of VA respondents and their relationship to the decedent, by module. (DOCX 13 kb)
Additional file 3: Odds ratios from logistic regressions 1, 2 and 3 showing the effects of recall period on correct assignment for verbal autopsy pairs for adults only. Results of logistic regression models analyzing predictors of correct assignment for adults only. (DOCX 14 kb)

## Abbreviations

COD: Cause of death
CRVS: Civil registration and vital statistics
GS: Gold standard
NHMRC: Australian National Health and Medical Research Council
PHMRC: Population Health Metrics Research Consortium
VA: Verbal autopsy
VAI: Verbal autopsy instrument
WHO: World Health Organization
