# Supplementary material for: What is the optimal recall period for verbal autopsies? Validation study based on repeat interviews in three populations
Source: Popul Health Metr. 2016 Oct 18;14:40. doi: 10.1186/s12963-016-0105-1 (PMC5101705; doi:10.1186/s12963-016-0105-1)
Supplement: Additional file 1: — List of target causes for the VA for adults, children and neonates. (DOCX 12 kb) [file 12963_2016_105_MOESM1_ESM.docx]

**Additional file 1. List of target causes for the VA**

| **Adult Cause List** | **Child Cause List** | **Neonate Cause List** |
| --- | --- | --- |
| AIDS | AIDS | Birth asphyxia |
| Asthma | Bite of Venomous Animal | Congenital malformation |
| Bite of Venomous Animal | Diarrhea/Dysentery | Meningitis/Sepsis |
| Breast Cancer | Drowning | Pneumonia |
| Cervical Cancer | Encephalitis | Preterm Delivery |
| Cirrhosis | Falls | Stillbirth |
| Colorectal Cancer | Fires |  |
| COPD | Hemorrhagic fever |  |
| Diabetes | Malaria |  |
| Diarrhea/Dysentery | Measles |  |
| Drowning | Meningitis |  |
| Epilepsy | Other Cancers |  |
| Esophageal Cancer | Other Cardiovascular Diseases |  |
| Falls | Other Defined Causes of Child Deaths |  |
| Fires | Other Digestive Diseases |  |
| Homicide | Other Infectious Diseases |  |
| Acute Myocardial Infarction | Pneumonia |  |
| Leukemia/Lymphomas | Poisonings |  |
| Lung Cancer | Road Traffic |  |
| Malaria | Sepsis |  |
| Maternal | Violent Death |  |
| Other Cardiovascular Diseases |  |  |
| Other Infectious Diseases |  |  |
| Other Injuries |  |  |
| Other Non-communicable Diseases |  |  |
| Pneumonia |  |  |
| Poisonings |  |  |
| Prostate Cancer |  |  |
| Renal Failure |  |  |
| Road Traffic |  |  |
| Stomach Cancer |  |  |
| Stroke |  |  |
| Suicide |  |  |
| TB |  |  |
