# Supplementary material for: What is the optimal recall period for verbal autopsies? Validation study based on repeat interviews in three populations
Source: Popul Health Metr. 2016 Oct 18;14:40. doi: 10.1186/s12963-016-0105-1 (PMC5101705; doi:10.1186/s12963-016-0105-1)
Supplement: Additional file 2: — Summary of the sample size of VA respondents and their relationship to the decedent, by module. (DOCX 13 kb) [file 12963_2016_105_MOESM2_ESM.docx]

Additional file 2. Summary of the sample size of VA respondents and their relationship to the decedent, by module

| **Relationship** | **Module** | | | |
| --- | --- | --- | --- | --- |
|  | Adult | Child | Neonate | Total |
| Child | 883 | 0 | 0 | 883 |
| Extended family | 209 | 0 | 0 | 209 |
| Father | 101 | 148 | 86 | 335 |
| Mother | 331 | 352 | 507 | 1,190 |
| Other | 37 | 198 | 147 | 382 |
| Sibling | 345 | 0 | 0 | 345 |
| Spouse | 882 | 0 | 0 | 882 |
| Total | 2,788 | 698 | 740 | 4,226 |
