# Supplementary material for: What is the optimal recall period for verbal autopsies? Validation study based on repeat interviews in three populations
Source: Popul Health Metr. 2016 Oct 18;14:40. doi: 10.1186/s12963-016-0105-1 (PMC5101705; doi:10.1186/s12963-016-0105-1)
Supplement: Additional file 3: — Odds ratios from logistic regressions 1, 2 and 3 showing the effects of recall period on correct assignment for verbal autopsy pairs for adults only. Results of logistic regression models analyzing predictors of correct assignment for adults only. (DOCX 14 kb) [file 12963_2016_105_MOESM3_ESM.docx]

Additional file 3. **Odds ratios from logistic regressions 1, 2 and 3 showing the effects of recall period on correct assignment for verbal autopsy pairs for adults only.**

| **Covariates** | **Regression 1 (adults only)** | **Regression 2 (adults only)** | **Regression 3 (adults only)** |
| --- | --- | --- | --- |
|  | **OR (95% CI)** | **OR (95% CI)** | **OR (95% CI)** |
|  | **N=1394** | **N=1394** | **N=1394** |
| Recall period (months) | 0.993 (0.975, 1.011) |  |  |
| Recall period (months^2^) | 1.000 (1.000, 1.001) |  |  |
| Recall period 0-2 months (reference) |  |  |  |
| Recall period 3-11 months |  | 1.002 (0.865, 1.160) |  |
| Recall period ≥ 12 months |  | 0.891 (0.755, 1.053) |  |
| Recall period ≥ 3 months |  |  | 0.955 (0.848, 1.074) |
| Andhra Pradesh (reference) |  |  |  |
| Manila | 1.303 (0.986, 1.720) | 1.324 (1.001, 1.751) | 1.299 (0.983, 1.715) |
| Bohol (1) | 1.149 (0.892, 1.479) | 1.179 (0.912, 1.524) | 1.146 (0.891, 1.475) |
| Bohol (2) | 1.646 (1.268, 2.138) | 1.714 (1.351, 2.173) | 1.643 (1.310, 2.061) |
